# 3D-printed hemipelvic prosthesis combined with a dual mobility bearing in patients with primary malignant neoplasm involving the acetabulum: clinical outcomes and finite element analysis

**DOI:** 10.1186/s12893-022-01804-8

**Published:** 2022-10-06

**Authors:** Miao Wang, Tianze Liu, Changli Xu, Chang Liu, Bo Li, Qiujian Lian, Tongjiang Chen, Suchi Qiao, Zhiwei Wang

**Affiliations:** 1 Department of Orthopedics, Changhai Hospital, Naval Medical University (Second Military Medical University), Shanghai, 200433 People’s Republic of China; 2 Department of Orthopedics, The Third Affiliated Hospital, Naval Medical University (Second Military Medical University), 700 North Moyu Road, Jiading District, Shanghai, 201805 People’s Republic of China; 3 Department of Orthopedics, The 900th Hospital of Joint Logistic Support Force, 156 North Xi-er Huan Road, Gulou District, Fuzhou, 350025 Fujian People’s Republic of China

**Keywords:** Pelvic, Bone neoplasm, Prosthesis design, Dual mobility, Finite element analysis

## Abstract

**Background:**

Limb salvage reconstruction for pelvic tumors, especially periacetabular tumors, is challenging. We combined the use of dual mobility bearing and 3D-printed hemipelvic prosthesis to improve function and reduce the probability of complications after hemi-pelvic resection in patients with primary acetabular malignancy. The purpose of this study was to evaluate the efficacy and safety of this combination.

**Methods:**

Between October 2011 and May 2021, 11 patients with malignancies involving the acetabulum received hemipelvic replacement with a 3D-printed prosthesis and dual mobility bearing. Follow‐up of postoperative survival, complications, and Musculoskeletal Tumor Society 93 (MSTS-93) lower limb functional scores were carried out. A finite element model of the postoperative pelvis was developed and input into the finite element analysis software. The Von Mises equivalent stress formula was used to analyze the stress distribution of each part of the pelvis under one gait cycle and the stress distribution at different angles of the hip joint.

**Results:**

By the last follow-up, 9 of the 11 patients (81.8%) were still alive, and 2 patients had local tumor recurrence. The complications including 1 deep infection and 1 dislocation of the artificial joint. Excluding 1 amputation patient, the average score of the remaining 8 patients at the last follow-up was 21.4/30 (71.3%) on the MSTS-93. In the reconstructed pelvis, stress distributions were concentrated on the junction between hemipelvic prosthesis and screw and iliac bone on the resected side, and between femoral prosthesis stem and femoral bulb, while the stress of polyethylene lining was small. Before impact, the polyethylene lining will rotate at a small angle, about 3°. The inner stress of polyethylene liner is greater than the outer stress in all conditions. The polyethylene liner has no tendency to slide out.

**Conclusion:**

Pelvic tumor resection and reconstruction using 3D-printed hemipelvic prosthesis combined with dual mobility bearing was an effective treatment for pelvic tumors. Our patients achieved good early postoperative efficacy and functional recovery. The dual mobility bearing is beneficial to prevent dislocation, and the mechanical distribution and wear of the prosthesis are acceptable.

**Supplementary Information:**

The online version contains supplementary material available at 10.1186/s12893-022-01804-8.

## Background

For patients with a pelvic neoplasm involving the acetabulum, reconstructing the pelvis after tumor resection may be a challenge [1]. The development of prostheses has enabled many options in pelvic reconstruction, such as the saddle prosthesis [2], modular prosthesis [3], and three-dimensional (3D)-printed prosthesis [4]. However, in all of these options, there remain high rates of complications, and postoperative joint function may be poor. The overall postoperative complication rate after hemipelvectomy for treatment of malignant neoplasms of the pelvis is approximately 50%, and hip dislocation is one of the most common complications [5, 6]. Because dual mobility components have demonstrated good anti-dislocation effects in hip arthroplasty [7], we speculated that combining a dual mobility bearing with a 3D-printed hemipelvic prosthesis may reduce the risk of dislocation after hemipelvectomy.

Finite element analyses have been frequently used in human biomechanics research. Compared with clinical trials or mechanical experiments of the body, finite element analysis more easily provides both dynamic and static response information under a variety of loading and boundary conditions [8]. Several previous studies have conducted finite element analyses to assess pelvic prostheses and have shown the effectiveness and feasibility of using different prosthetic construction schemes [9, 10].

In the present study, we combined dual mobility components with a 3D-printed, customized, hemipelvic prosthesis that originated from one of our patents. We retrospectively analyzed the short-term outcomes of 11 patients with pelvic neoplasm whose pelvis was reconstructed using this prosthesis at our medical institution. Finite element analysis was conducted to assess the prosthesis in the only patient to experience postoperative dislocation. To our knowledge, this is the first report assessing the efficacy of a customized hemipelvic prosthesis with dual mobility components.

## Methods

This study was performed in accordance with the Helsinki Declaration of 1975, as revised in 1983 and was authorized by the ethics committee of Changhai Hospital. Written informed consent to participate in this study was obtained from each patient.

### Patients

We retrospectively analyzed patients with pelvic neoplasms who underwent surgery at the Changhai Hospital affiliated to the Naval Medical University (Second Military Medical University) from 2017 to 2021. The inclusion criteria were as follows: (1) The tumor involved the acetabulum (Enneking system stage II [11]), requiring functional reconstruction. (2) No implant contraindication. (3) Safe tumor surgical resection margins were achieved intraoperatively. (4) For patients with osteosarcoma or Ewing sarcoma, the tumors were sensitive to neoadjuvant chemotherapy. (5) Patient anesthesia score was not higher than II using the American Society of Anesthesiology Physical Status Classification System [12]. (6) The patient had an estimated survival time before surgery of > 6 months. (7) The patient agreed to participate in the trial and provided written informed consent.

The exclusion criteria for this study were as follows: (1) Distant metastasis occurring before surgery. (2) The lesions in the pelvis were metastatic. (3) Patients with other medical conditions that might affect life expectancy. (4) Patients who had other neurological disorders that might affect postoperative function. (5) Patients whose tumors could not be completely removed during surgery. (6) Postoperative follow-up was < 6 months.

### Prosthesis design

**Fig. 1:**
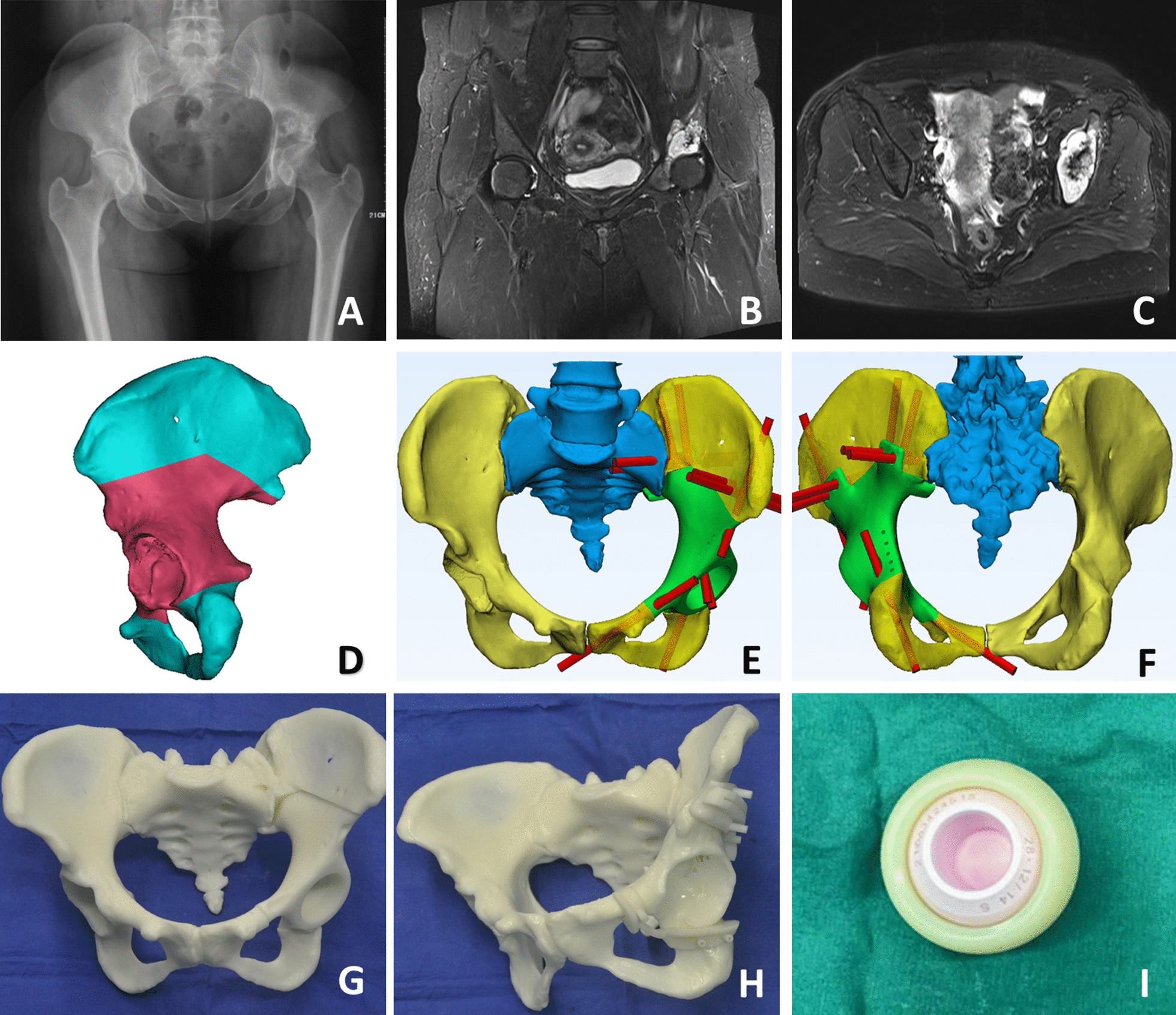
Preoperative planning for patient 11, a 42-year-old female with left pelvic chondrosarcoma. **A**–**C** Radiograph and magnetic resonance imaging scans showing tumor involvement in pelvic regions I and II. **D**–**F** Computer simulated tumor resection and reconstruction. **G**, **H** 3D‐printed 1:1 ratio lesion model and osteotomy. **I** Image of a dual mobility bearing

All patients underwent X-Ray, 3D computed tomography (CT), and magnetic resonance imaging (MRI) of the pelvis to determine the nature, size, invasion degree, and scope of the tumor resection (Fig. 1A–C). The CT scan of the pelvis (thickness of 1 mm) was saved in DICOM format and input into Mimics software (Materialise Company, Belgium) for data segmentation to build virtual 3D models of the pelvis. The digital model was used to simulate the osteotomy location and to determine the location of the stereoscopic osteotomy. The tumor-free resection margin was set as 15 mm for chondrosarcoma, fibrosarcoma, and giant cell tumor of bone, and as 30 mm for osteosarcoma and Ewing sarcoma. After the pelvic prosthetic construction program was completed (Fig. 1D–F), the data were sent to the prosthesis manufacturers (Chun Li, Ltd. Beijing, China), which provided a 3D-printed 1:1 ratio lesion model (Fig. 1G, H). The design procedures took 2 days. The 3D-printing fabrication, post-processing, and delivery took 7 days.

The prosthesis consisted of a 3D-printed hemipelvic prosthesis (Chun Li Ltd., Beijing, China) with a dual mobility polyethylene lining, a femoral head, and a femoral stem (LINK GMBH, Germany). The design and manufacturing cost was approximately US $15,000. The surface of the contact area between the hemipelvic prosthesis and the residual bone was 3D-printed tantalum trabecular bone to ensure bone ingrowth postoperatively. The femoral stem was a cementless prosthesis, and the femoral head prosthesis was made of ceramic or metal. During the operation, a press-fit device was used to press the customized femoral head into the matching polyethylene lining. The metal cup and its surface metal liner—a vitamin E-rich highly cross-linked polyethylene lining—and femoral head prosthesis constituted the dual mobility bearing (Fig. 1I). The outer surface of the polyethylene lining was connected with the side of the hemipelvic prosthesis to form a movable surface, while the inner surface was movably connected to the femoral head. The limit was added at the joint between the lining and the femoral head, and the interference was approximately 0.15 to 0.20 mm on one side.

### Surgical techniques

#### Preoperative preparation

**Fig. 2:**
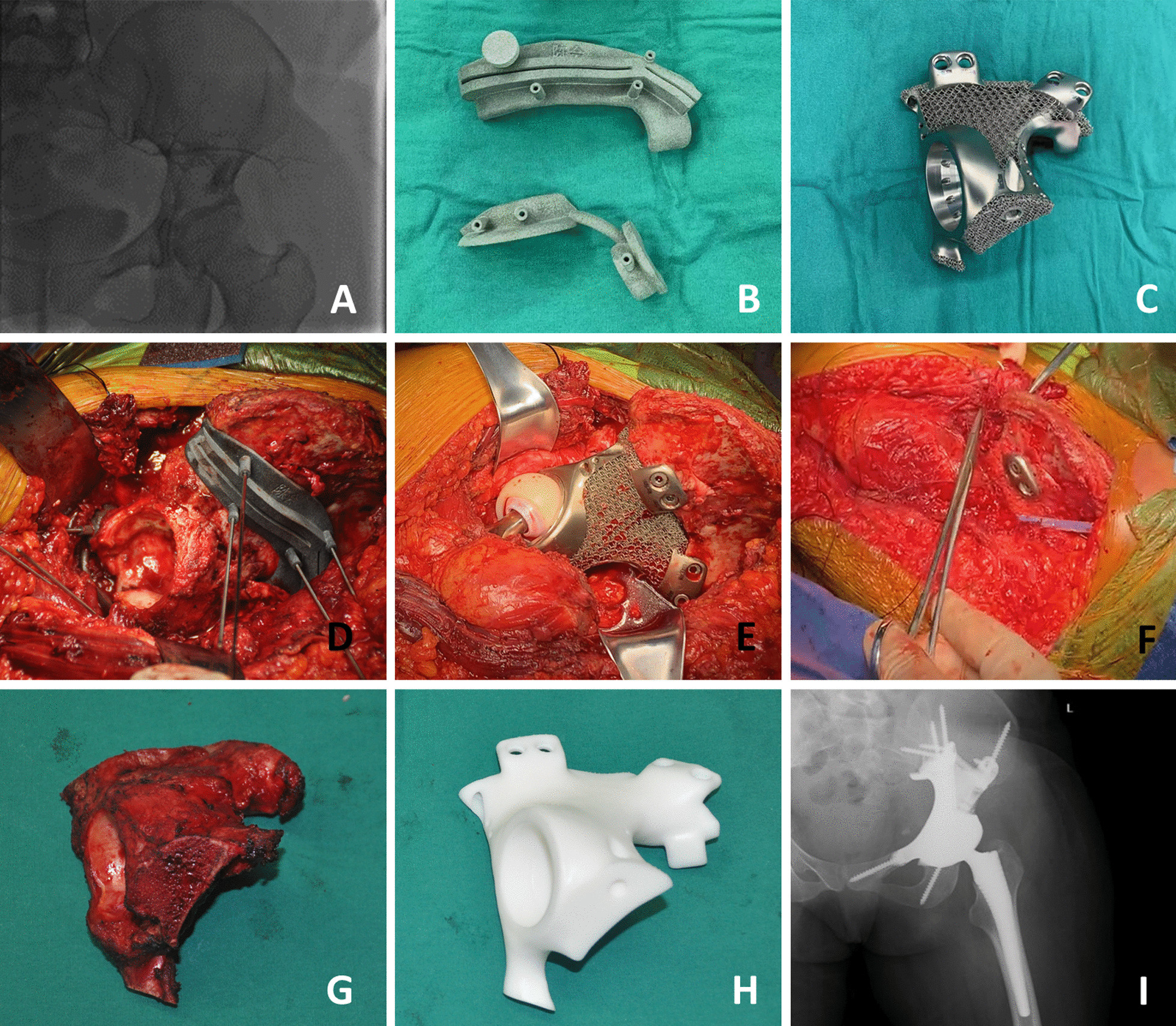
Surgical resection and implantation procedure. **A** Preoperative tumor vascular embolization. **B** Customized osteotomy template. **C** 3D-printed hemipelvic prosthesis. **D** Intraoperative osteotomy performed with the aid of the customized osteotomy template. **E** Implantation of the 3D-printed hemipelvic prosthesis. The reconstructed joint did not dislocate even in an extreme position. **F** Suturing of the surrounding soft tissue. **G**, **H** Comparison of the intact hemipelvic bone and the 3D-printed hemipelvic prosthesis. **I** Radiograph obtained 6 months after the procedure

All the patients were evaluated preoperatively using the Enneking staging system [13]. Needle biopsy or open biopsy was used to identify the pathological type of tumor. All patients were required to undergo positron emission tomography/computerized tomography (PET/CT) to determine whether the tumor had metastasized. In addition, patients underwent contrast-enhanced MRI and contrast-enhanced CT within 7 days before radical resection to confirm the invasion area and blood supply of the tumor. On the day before surgery, except for one patient who was allergic to contrast media, all patients received tumor embolization (Fig. 2A). In addition, patients with osteosarcoma and Ewing sarcoma received neoadjuvant chemotherapy before surgery.

#### Surgical procedure

The patients underwent surgery in a lateral position, on the contralateral side, to enable forward and backward movements of the leg during the operation. Using the preoperative plan, the osteotomy template was placed where the osteotomy was expected (Fig. 2B, C). After pelvic osteotomy and complete tumor resection, the surgical area was cleaned with hydrogen peroxide and hypotonic water. The components of the 3D-printed hemipelvic prosthesis (including the pelvic components, the femoral stem prosthesis, and the dual mobility components) were placed according to the preoperative design. The space between bone and prothesis was filled with autologous cancellous bone. After reduction, the hip joint was confirmed during surgery not to become dislocated even in an extreme position. Finally, soft tissue reconstruction was performed: the piriformis muscle and other severed external rotator stumps were reconstructed into several strands, which were sutured with nonabsorbable sutures to the reserved holes in the hemipelvic prosthesis. The stumps of the abductor and adductor muscles were sutured to the abdominal muscles or fixed in the reserved holes of implant. An additional figure file shows this in more detail (Additional file 1: Figure S1). An antibiotic was used preventively during surgery according to the guidelines (Fig. 2D–I).

#### Postoperative recovery and follow-up

Physical therapy (e.g., ankle pump exercise, isometric contraction of lower extremity muscles) was started at the first day after surgery to prevent venous thrombosis. The systematic rehabilitation exercises were carried out in rehabilitation hospitals. From 1 month after the operation, patients walked with the aid of a walker and performed exercises to enhance the stability of the muscles around the hip and proprioception. At 3 months after surgery, walking with a cane without crutches and performing further functional exercises. An additional flow chart file shows this in more detail (Additional file 2: Figure S2).

The patients were followed up regularly every 3 to 6 months. A pelvic radiograph and a chest CT scan were obtained at 3, 6, and 12 months after surgery. Limb function, complications, and whether the tumor had recurred or metastasized were the focus of the follow-up. The Musculoskeletal Tumor Society 93 (MSTS-93) scoring system [14] was used for evaluating the postoperative function of each patient at 6 and 12 months after surgery. The MSTS-93 scoring system includes six variables: pain, function, emotional acceptance, support, walking ability, and gait. Each variable was assessed on a 5-point scale, with a maximum score of 30. The final result was calculated as a percentage. SPSS 21.0 (IBM Inc, IL, USA) was used for statistical analysis.

### Finite element analysis

The only patient to experience recurrent artificial joint dislocation after surgery was selected for finite element analysis, and the model of the reconstructed pelvis and surrounding ligaments was imported into Altair HyperMesh. We executed the modules Seed Part, Mesh Control, and Mesh Part in sequence to complete the Mesh division of the reconstructed pelvic model, which totaled 113,952 nodes and 477,578 Mesh units. The sacroiliac articular cartilage and pubic symphysis were divided into eight IsoMesh Hex elements and the remaining four elements into tetrahedral Mesh elements (Tet Mesh Tet). The ligaments were set as two-node line Spring elements (1D Spring). The obtained BDF files were imported into the finite element pre-processing software MSC Nastran (Patran, 2019) to set finite element mesh properties, including defined material parameters, applied loads, boundary condition constraints, and working condition settings. The implant material property values were provided by Chun Li Ltd. and LINK GMBH. Relevant material coefficients were obtained from previous studies [15, 16] (Additional file 3: Table S1). Calculations and data processing were performed in the finite element post-processing software MSC Nastran. The strength of the periacetabular muscles was not considered because it is greatly affected by widespread resection and can not be calculated accurately. The von Mises equivalent peak stress was used to analyze the mechanical distribution of the pelvis. For standard gait simulation, we applied a force of 500 N to approximate the human weight above the pelvis and set an axial force of 2000 N along the femoral prosthesis for the extreme position test.

## Results

### Patient characteristics

A total of 11 patients (6 men and 5 women) were included in the study (Table 1). The mean age at first diagnosis was 45 years (range 24–57 years). The tumors in all patients were primary, including five patients with chondrosarcoma, two patients with osteosarcoma, two patients with giant cell tumor of bone, one patient with fibrosarcoma, and one patient with Ewing sarcoma (Table 1).

**Table 1:** Demographic and clinical characteristics and follow-up results of patients who underwent reconstruction following tumor resection

Case no	Age, year	Sex	Follow-up, months	Diagnosis	Staging^a^	Resection type^b^	Blood loss, mL	Operation time, hours	Status	Outcome	Pain^c^	Function^c^	Emotional acceptance^c^	Support^c^	Walking ability^c^	Gait^c^	Total score (%)^c^
1	34	F	12	Ewing sarcoma	IIB	I + II + III	3500	10	dec	Local recurrence and distant metastasis (lung)	NA	NA	NA	NA	NA	NA	NA
2	54	F	50	Chondrosarcoma	IIB	II	4500	9	ned	Intact reconstruction	5	4	5	3	3	3	23 (76.7)
3	43	M	44	Chondrosarcoma	IIB	II + III	6000	9	ned	Intact reconstruction	4	3	4	3	3	3	20 (66.7)
4	24	M	4	Osteosarcoma	IIB	II + III	3000	6	dec	Complications of chemotherapy	NA	NA	NA	NA	NA	NA	NA
5	56	M	40	Chondrosarcoma	IIB	I + II	3000	8	ned	Intact reconstruction	4	4	5	4	4	4	25 (83.3)
6	27	F	36	Ewing sarcoma	IIB	I + II + III	3500	6.5	ned	Intact reconstruction	3	3	4	3	3	3	19 (63.3)
7	57	M	30	Osteosarcoma	IIB	I + II + III	3000	6	ned	Amputation for local recurrence	NA	NA	NA	NA	NA	NA	NA
8	53	M	24	Fibrosarcoma	IIB	I + II	3000	4.5	ned	Repeated dislocation	3	3	4	3	3	2	18 (60)
9	41	F	22	Giant cell tumor	IIA	II	1500	4.5	ned	Intact reconstruction	4	4	5	4	4	4	25 (83.3)
10	63	M	14	Chondrosarcoma	IIA	II	2000	5.5	ned	Intact reconstruction	4	3	4	3	3	3	20 (66.7)
11	43	F	10	Chondrosarcoma	IIA	II	1500	4	ned	Intact reconstruction	4	3	5	3	3	3	21 (70)

*F* female, *M* male, *dec* deceased, *ned* no evidence of disease, *NA* not assessed

^a^Staging according to the Enneking system

^b^Resection type indicates pelvic regions involved

^c^The Musculoskeletal Tumor Society 93 scoring system was used for evaluating postoperative function; each of the six variables was assessed on a 5-point scale, with a maximum total score of 30

For patients with osteosarcoma or Ewing sarcoma, neoadjuvant chemotherapy was given preoperatively. The results of magnetic resonance imaging scans indicated that the tumor volume was reduced and that the boundary was clearer in all patients after receiving neoadjuvant chemotherapy.

### Oncological outcomes

As of March 1, 2022, two patients had died. One patient with osteosarcoma developed hematological complications during chemotherapy 4 months after surgery and died at another hospital. One patient with Ewing sarcoma died 12 months after surgery due to tumor recurrence and distant metastasis (Table 1).

Among 11 patients, two patients experience local tumor recurrence. For the patient with osteosarcoma who was alive as of March 1, 2022, the tumor recurred 18 months after surgery. After being comprehensively evaluated, the patient underwent hemipelvic amputation and the prosthesis was removed. At the last follow-up, there was no sign of tumor recurrence in this patient.

### Functional recovery after surgery

The MSTS-93 scores of eight patients were included in the study (the scores for two patients who were deceased and for one patient who underwent hemipelvic amputation were excluded). The mean postoperative follow‐up was 30 months (range 10–50 months). The mean MSTS-93 score for eight patients was 21.38 (71.3%; range 60.0–83.3%). There were no differences in MSTS-93 scores among three patients with tumors that were limited (Enneking stage II) and five patients with tumors involving other areas (t = 0.451, p > 0.05).

The MSTS-93 scores for each of the six variables were as follows (5 points maximum per variable). (1) Pain: Pain was relieved in all patients after surgery, with a mean score of 3.88 points (77.5%). (2) Function: The mean score was 3.38 points (67.5%). The function of the affected limb was nearly unlimited in three cases, and slightly limited in five cases. (3) Emotional acceptance: The mean score was 4.50 points (90%). Most patients were satisfied with their postoperative recovery. (4) Support: The mean score was 3.25 points (65%). Two patients were able to walk without support. (5) Walking ability: The mean score was 3.25 points (62.5%). The ability to walk was partially restricted in six patients. (6) Gait: The mean score was 3.13 points (62.5%). Gait changes were observed in all eight patients (Table 1).

### Complications

One patient experienced deep tissue infection at the surgical site 3 months after surgery. The infection resolved after wound debridement and antibiotic treatment.

**Fig. 3:**
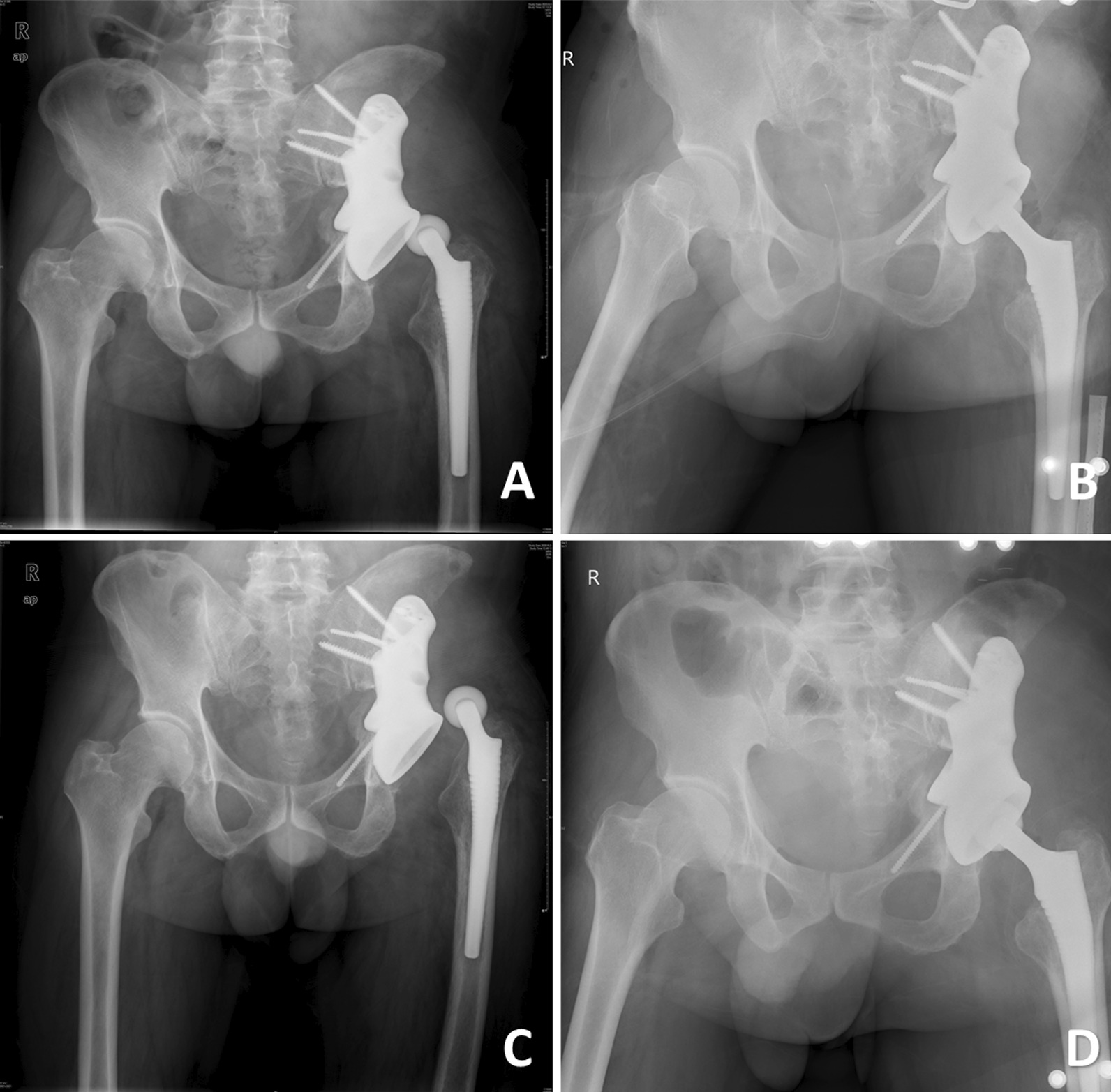
Radiograph images of a patient with recurrent dislocation. **A** Radiograph image obtained before the open reduction. **B** Radiograph image obtained after the reduction. **C** Radiograph image obtained before placement of the polyethylene liner and femoral head. **D** Radiograph image obtained after placement of polyethylene liner and femoral head

One patient experienced recurrent hip dislocation in the reconstructed hip joint. The first dislocation occurred 5 months after surgery. A closed reduction was performed at another hospital. The second dislocation occurred 7 months after surgery. The hip component prosthesis was openly reduced. The last dislocation to date occurred 11 months after the radical resection. The polyethylene lining and the ceramic femoral head were replaced (Fig. 3).

### Finite element analysis of the patient with dislocation

**Fig. 4:**
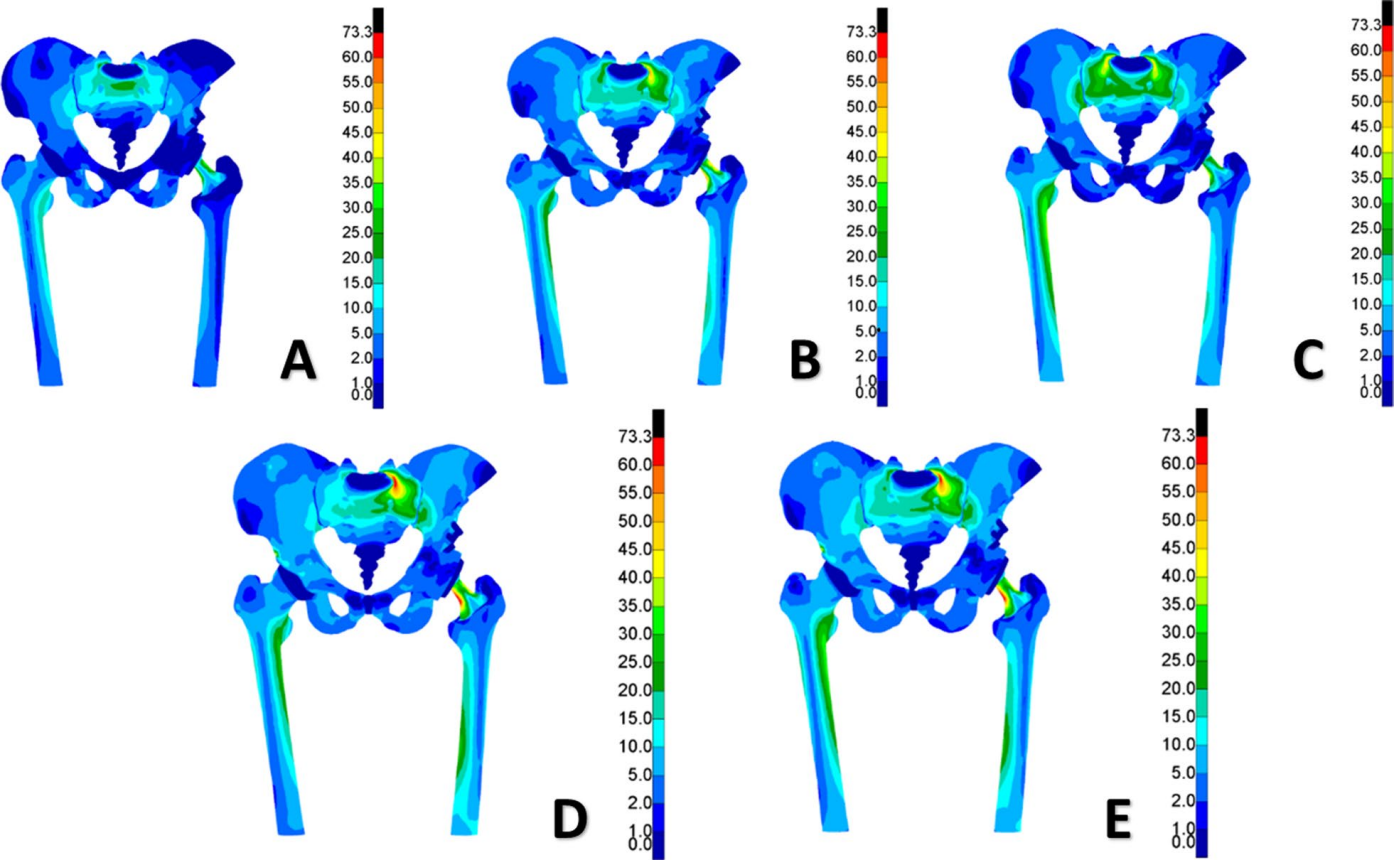
Distribution of the stress in the postoperative pelvis and hip joint at different phases of the gait. **A** Heel on the ground. **B** Toe on the ground. **C** Midstance. **D** Heel off the ground. **E** Toe off the ground

**Fig. 5:**
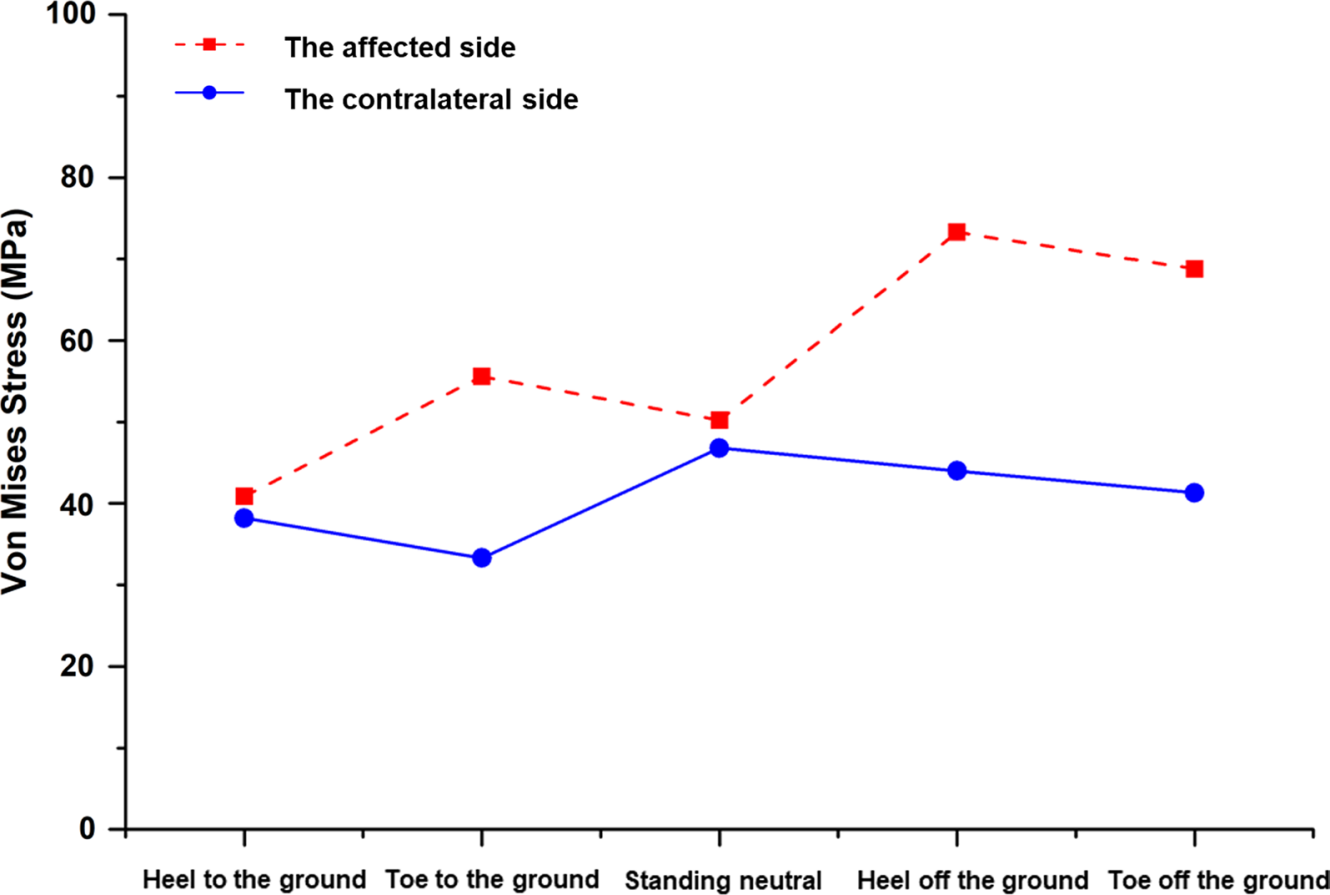
Comparison of cortical bone stress on the affected side vs the contralateral side at different gait phases

**Fig. 6:**
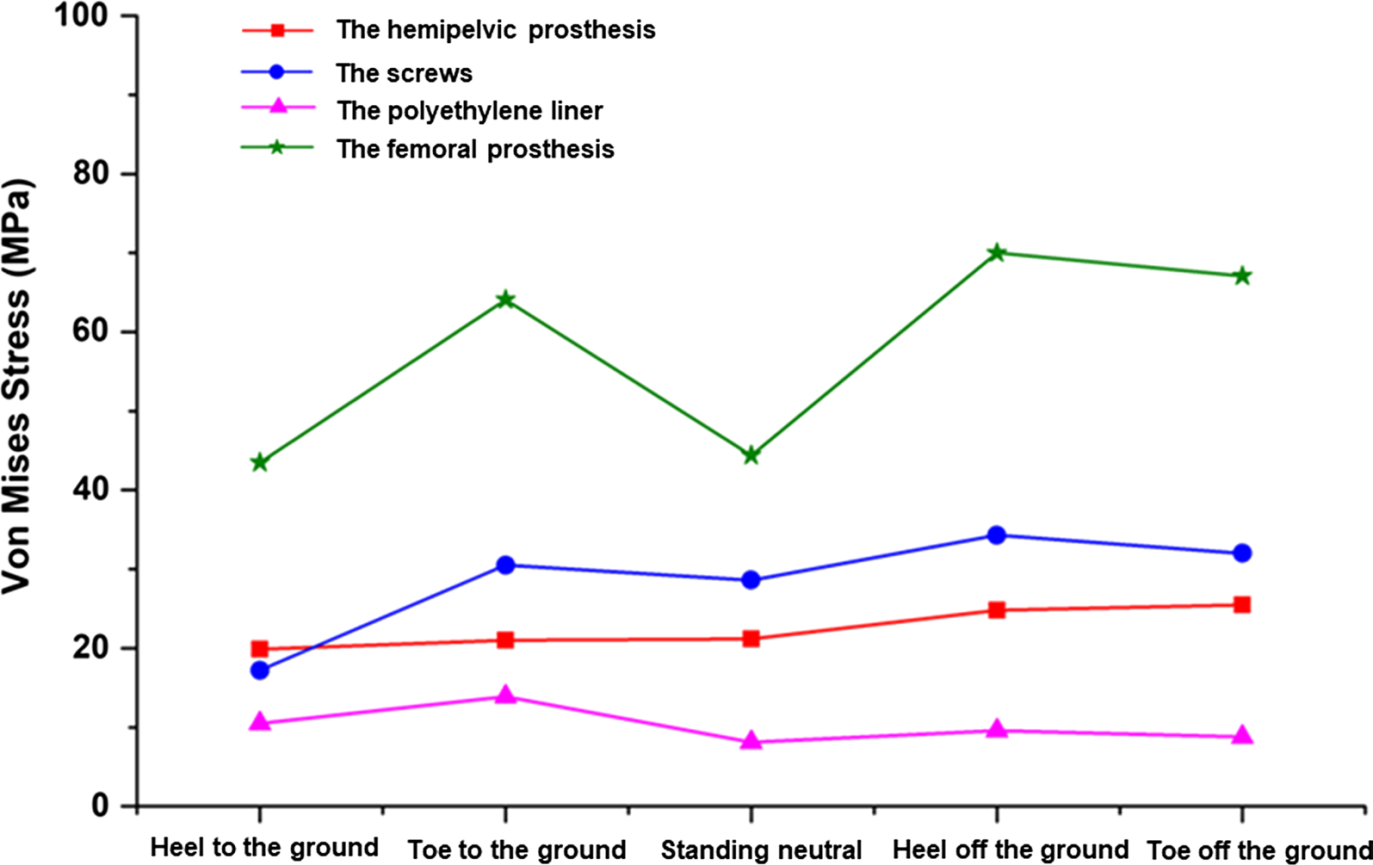
Distribution of stress across various components of the prosthesis during different phases of the gait

The distribution of the stresses on the affected side of the postoperative pelvis (including the cortical bone, hemipelvic prosthesis, screw, polyethylene liner, and femoral prosthesis) were compared with those on the contralateral side and with a normal pelvis at different gait phases (Fig. 4, Table 2). For a normal gait, the stress was mainly distributed at the fixation between the prosthesis and sacroiliac joint and the connection between the femoral prosthesis stem and the femoral ball, whereas the polyethylene lining bore less stress (Fig. 5). At the gait phase with the heel off the ground, the values for the stress of the pelvic cortical bone and prosthesis were higher than those for other gait phases (Fig. 6). The stress distribution on the affected side of the pelvis after surgery was similar to that of the contralateral and normal pelvis. However, the peak value of local stress on the affected side was higher than that of the contralateral side, and this was most obvious in the gait phase from the heel off the ground to the toe off the ground.

**Table 2:** Distribution of stress in the postoperative pelvis and hip joint at different phases of gait (MPa)

	Cortical bone of affected side pelvis	Cortical bone of unaffected side pelvis	Hemipelvic prosthesis	Screws	Polyethylene liner	Femoral prosthesis
Heel to the ground	40.9	38.2	19.9	17.2	8.5	43.5
Toe to the ground	55.6	33.3	21.0	30.5	10.9	64.1
Midstance	50.2	46.8	21.2	28.6	5.1	44.4
Heel off the ground	73.3	44.0	24.8	34.3	6.6	70.0
Toe off the ground	68.8	41.3	25.5	32.0	5.8	67.1

**Fig. 7:**
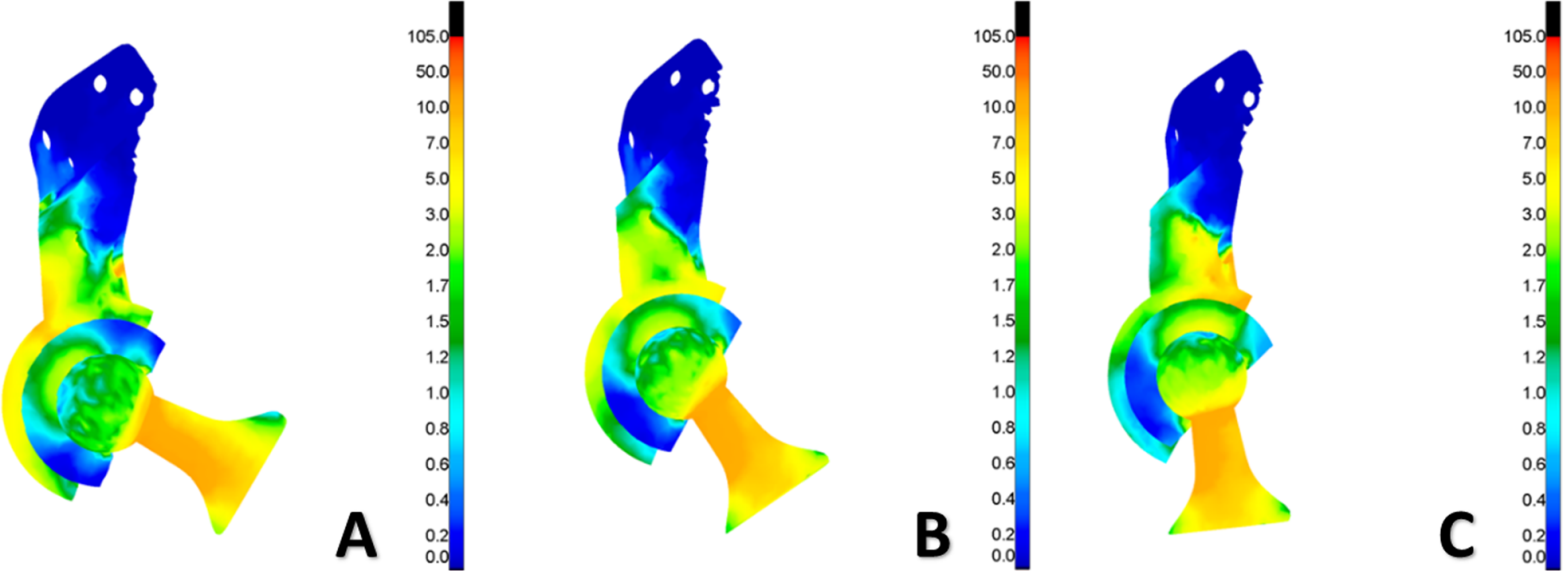
Distribution of stress in the hemipelvic prosthesis and dual mobility bearing at three different angles with a joint compressive force of 2000 N. **A** 0°; **B** 30°; **C** 65°

We investigated the cause of the hip dislocation using finite element analysis. The position of the femoral neck perpendicular to the polyethylene-lined prosthesis was defined as 0°. With the femoral prosthesis gradually flexed-adducted from the 0° position, as the flexion-adduction angle increased, the degree of compression of the femoral head prosthesis on the inner side of the polyethylene liner became weaker, and the compression position changed with the angle. For all positions, the stress on the inner side of the polyethylene liner was higher than that on the outer side. When the movement reached 38°, the area where the polyethylene liner contacted the femoral prosthesis stem was squeezed, and the stress was concentrated. The lining experienced more pronounced frictional movement in the acetabulum. At a rotation less than 38°, the polyethylene liner showed a slight angle of movement, a change within about 3°; however, when the femoral prosthesis was moved to a maximum rotation of roughly 65°, the polyethylene liner was displaced by approximately 14°. In this position, the concentration of the polyethylene lateral stress was located in the range of the metal acetabulum, and the polyethylene lining did not slide out (Fig. 7).

## Discussion

In this study, we retrospectively analyzed the outcomes of patients with primary malignant neoplasm of the acetabulum requiring hemipelvectomy and receiving a 3D-printed hemipelvic prosthesis with a dual mobility bearing, and we evaluated the safety and efficacy of the prosthesis. Our findings indicated that the prothesis was safe and feasible and provided good clinical and functional outcomes for most patients.

To our knowledge, studies assessing the application of a dual mobility bearing in hemipelvectomy and reconstruction are limited and the results are inconsistent. In a study by Philippeau et al., a dual mobility bearing was used to reconstruct the pelvis after tumor resection in 71 patients [17]. The outcomes demonstrated that this design prevented total artificial joint dislocation in some patients, and some patients had relatively good postoperative function. However, this design failed to prevent hip dislocation in patients with acetabular and abductor muscles/innervation involvement [17]. In another study, a dual mobility cup was combined with a LUMiC^®^ endoprosthesis to reconstruct the pelvis after periacetabular tumor resection [18]. The risk of hip dislocation was lower in reconstructions with the dual mobility cup (1 of 24, 4%) than in those without. However, the LUMiC^®^ endoprosthesis requires sufficient ilium for fixation. For patients with extensive tumor invasion, this prosthesis may not be suitable. The size of the 3D-printed customized hemipelvic prosthesis can be individually designed and is not theoretically limited by the extent of tumor invasion. Thus, we hypothesized that the combination of the 3D-printed hemipelvic prosthesis and a dual mobility bearing would provide a larger scope for application than the LUMiC^®^ endoprosthesis, better postoperative function, and fewer patients with hip dislocation. To our knowledge, no study has reported on this specific combination.

Of 11 patients in our study, one patient died and one patient underwent hemipelvic amputation owing to tumor recurrence, which is consistent with previously reported outcomes [5]. Excluding those two patients, the mean postoperative MSTS-93 score in our study was 21.4 (71.3%), which was similar to previous reports for patients receiving a custom-made prosthesis [3, 4, 19–28] (Table 3). Our study showed that the dual mobility components enabled the reconstructed hip joint to have a large range of motion, reduced the risk of dislocation, and helped patients achieve good functional recovery after engaging in short-term rehabilitation exercises. For example, the total MSTS-93 score of a patient only 10 months after surgery reached 21 (70%), a score not attained in previous studies. Despite changes in gait for all patients in the present study, most of them were satisfied with the overall limb function after surgery. Patients with ideal recovery could perform extreme movements, such as squatting, Patrick’s test. Notably, in contrast to previous studies, in the present study, there was no significant difference in functional outcomes between patients who underwent acetabulum reconstruction alone and those who underwent reconstruction of multiple areas involving the acetabulum [28]. Our outcomes suggested that patients with extensive resection and reconstruction of the pelvis may also achieve good postoperative function owing to the use of the dual mobility components. Although data from a larger sample are needed to support our findings, our results indicated that the combined use of a 3D-printed hemipelvic prosthesis and a dual mobility bearing warrants further follow-up and biomechanical analysis.

**Table 3:** Studies reporting hip reconstruction using a custom-made prosthesis or a prothesis with a dual mobility bearing

Study	Prosthesis	No. of patients	Follow-up duration, mean (range)	Functional outcome, mean MSTS-93 score (of 30 points maximum)	No. of patients with hip dislocation after procedure	Other complications (No. of patients)
Current study	3D Custom-made (with dual mobility bearing)	11	30 (10–50) months	21.4	1 of 11	Infection (1 of 11)
Wu [4]	3D Custom-made	28	32.2 (3–75) months	23	3 of 28	Superficial infection (6 of 28)
Peng [27]	3D Custom-made	5	30.3 (18–42) months	19.8	0 of 5	0 of 5
Wang [28]	3D Custom-made	13	27 (24–31) months	23	0 of 13	Delayed wound healing (2 of 13)
Holzapfel [25]	Custom-made	56	66 (1–270) months	18.4	11 of 56	Infection (14 of 56), delayed wound healing (10 of 56), loosening (3 of 56)
Sun [24]	Custom-made	16	36 (23–62) months	21.6	3 of 16	Delayed wound healing (6 of 16), prosthesis breakage (4 of 16), deep infection (1 of 16)
Guo [23]	Custom-made	18	41 (7–73) months	21.5	2 of 18	Delayed wound healing (2 of 18), deep vein thrombosis (1 of 18), loosening (1 of 18) and sciatic nerve palsy (1 of 18)
Jiaswal [22]	Custom-made	98	65 (2–33.5) months	Mean TESS score 17.8 of 30	19 of 98	Infection (30 of 98), loosening (3 of 98)and deep-vein thrombosis(7 of 98)
Dai [21]	3D Custom-made	10	34 (21–48) months	70% Good function	2 of 10	Deep infection (3 of 10), aseptic loosening (1 of 10)
Ozaki [20]	Custom-made	12	57 (26–77) months	Mean MSTS-87 score 11 of 30	1 of 12	Deep infection (3 of 12), loosening (3 of 12) and recurrence (4 of 12)
Abudu [19]	Custom-made	35	34 (12–312) months	21	6 of 35	Deep infection (9 of 35), aseptic loosening (2 of 35) and thromboembolism (1 of 35)
Bus [18]	Pedestal cup endoprosthesis; 24 with dual mobility cups	47	Minimum 24 months	21	10 of 47 (dual mobility cups, 1 of 24)	Deep infection (13 of 47), loosening (3 of 47)
Philippeau [17]	Dual mobility acetabular cups	71 (33 primary tumor, 38 with metastasis)	Primary, 3.3 years (0.6–7.1; metastasis, 1.25 years (0.2–7.9)	Metastasis, 20.4; Primary, 17.9	7 of 71	Deep infection (7 of 71), loosening (4 of 71)

*MSTS-93* Musculoskeletal Tumor Society 93, *TESS* Toronto Extremity Salvage Score

We also performed a finite element analysis for a pelvic model of one patient with postoperative dislocation to explore the biomechanical properties of the prosthesis. To our knowledge, this is the first finite element analysis of a 3D-printed hemipelvic prosthesis with a dual mobility bearing. The analysis indicated that with a normal gait and continuous hip flexion and adduction, the polyethylene liner of the dual mobility hemipelvic prosthesis bore less stress than at other positions, and the stress was mainly concentrated at the inner side of the polyethylene liner. Whereas our force analysis showed that the lateral interface of the polyethylene liner was less stressed, previous studies have suggested that with the same range of motion, the outer side of the polyethylene liner was more prone to wear than the inner side [26]. Highly cross-linked polyethylene has better anti-wear performance in a dual mobility bearing than does traditional polyethylene with a high molecular weight [16]. Given the particular requirements of patients with pelvic tumor, the highly cross-linked polyethylene may accommodate stress changes following tumor resection, alleviating concerns about the wear of the polyethylene lining. However, this assertion will require further biomechanical analysis.

We believe that hip dislocation in one patient in our study was not due to a design defect in the prosthesis. Before impact with the femoral neck, the polyethylene lining had 3° of fluidity and compensated for nearly 15° of movement angle at the extreme position, and the range of motion of the affected joint reached 130°. These values indicated that even when stress conditions were changed, compared with total hip arthroplasty, the fluidity of the dual mobility bearing in hemipelvic arthroplasty still existed. Thus, the range of motion was increased and effectively prevented dislocation. Even at an extreme limb position, the prosthesis showed no obvious tendency to dislocate. We hypothesize that after impingement of strong non-axial forces, the dislocation of the prosthesis may have caused the polyethylene liner to wear and deform, the dual mobility bearing lost its fluidity, and the dislocation occurred again after open reduction. After the polyethylene liner and the femoral head were replaced, the dislocation did not occur again despite weaker surrounding soft tissue, which indirectly supports this hypothesis.

Our findings showed that the distribution of stress on the affected side of the pelvis after surgery was similar to that of the contralateral pelvis and of a normal pelvis, but the peak local stress on the reconstructed side was higher than that on the unaffected side and also higher than previously reported data for total hip arthroplasty [29]. Although the structural stress on the reconstructed side was within its yield strength range and no loosening or fracture will occur in the short-term [30], the risk of postoperative loosening of the prosthesis is still greater than that of total hip arthroplasty. The service life of a prosthesis is also limited, which is a problem that requires resolution for the development of prostheses use in the future.

There are some limitations to this study. First, owing to low morbidity, the sample size was small and the follow‐up duration was short; thus, additional studies with longer-term follow-up durations are required. Second, owing to the rarity of complications, we conducted a finite element analysis for only one patient with hip dislocation. Further analyses in future studies of hip dislocation following pelvic tumor resection and reconstruction are required.

## Conclusions

The use of a 3D-printed hemipelvic prosthesis combined with a dual mobility bearing is an innovation. Most patients in the present study who received this combination had good functional outcomes. The results of a finite element analysis showed that the prosthesis had uniform force and a stable structure during a normal gait, with low risk of fracture or wear. The dual mobility bearing functioned normally during a normal walking gait and compensated for the range of motion at an extreme position. Thus, our findings indicated that a 3D-printed hemipelvic prosthesis combined with a dual mobility bearing is safe and feasible for use in patients with primary malignant neoplasms of the acetabulum requiring hemipelvectomy.

## Supplementary Information

**Additional file 1: Figure S1.** The figure file shows the proceeding of soft tissue reconstruction in more detail.

**Additional file 2: Figure S2.** The Flow chart shows our rehabilitation training plan.

**Additional file 3: Table S1.** The table shows the structural material coefficients we set in the finite element analysis.

## Data Availability

The data and materials are available from the medical records department of Changhai Hospital. The datasets used and analyzed during the current study are available from the corresponding author on reasonable request.

## Abbreviations

3D: Three-dimensional
MSTS-93: Musculoskeletal Tumor Society-93
CT: Computer tomograph
MRI: Magnetic resonance imaging
PET/CT: Positron emission tomography/computerized tomography
