# Supplementary figures and images for: 3D-printed hemipelvic prosthesis combined with a dual mobility bearing in patients with primary malignant neoplasm involving the acetabulum: clinical outcomes and finite element analysis
Source: BMC Surg. 2022 Oct 6;22:357. doi: 10.1186/s12893-022-01804-8 (PMC9541076; doi:10.1186/s12893-022-01804-8)

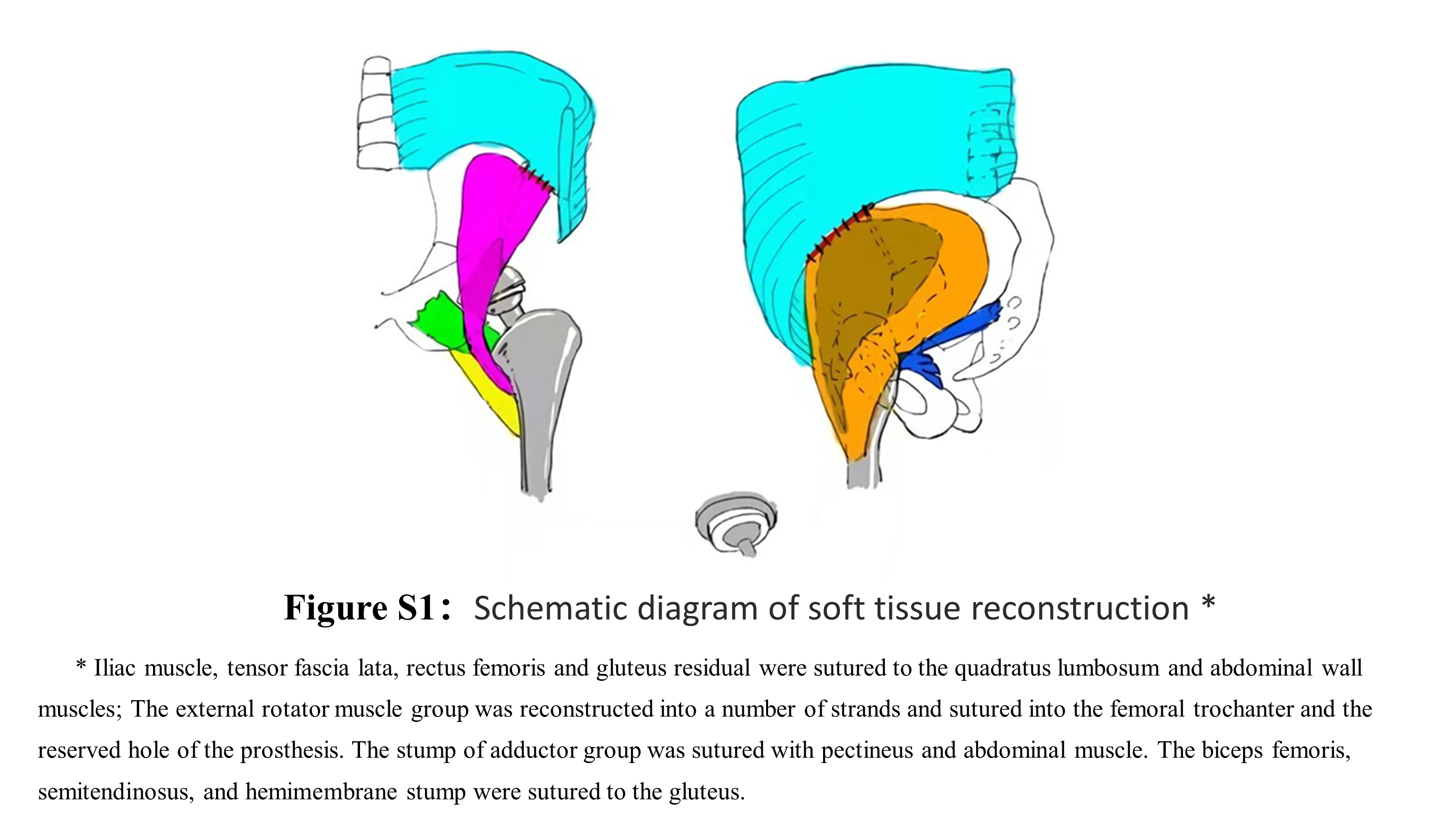

Supplement: Supplementary file 1 — Additional file 1: Figure S1. The figure file shows the proceeding of soft tissue reconstruction in more detail. [file 12893_2022_1804_MOESM1_ESM.tif]

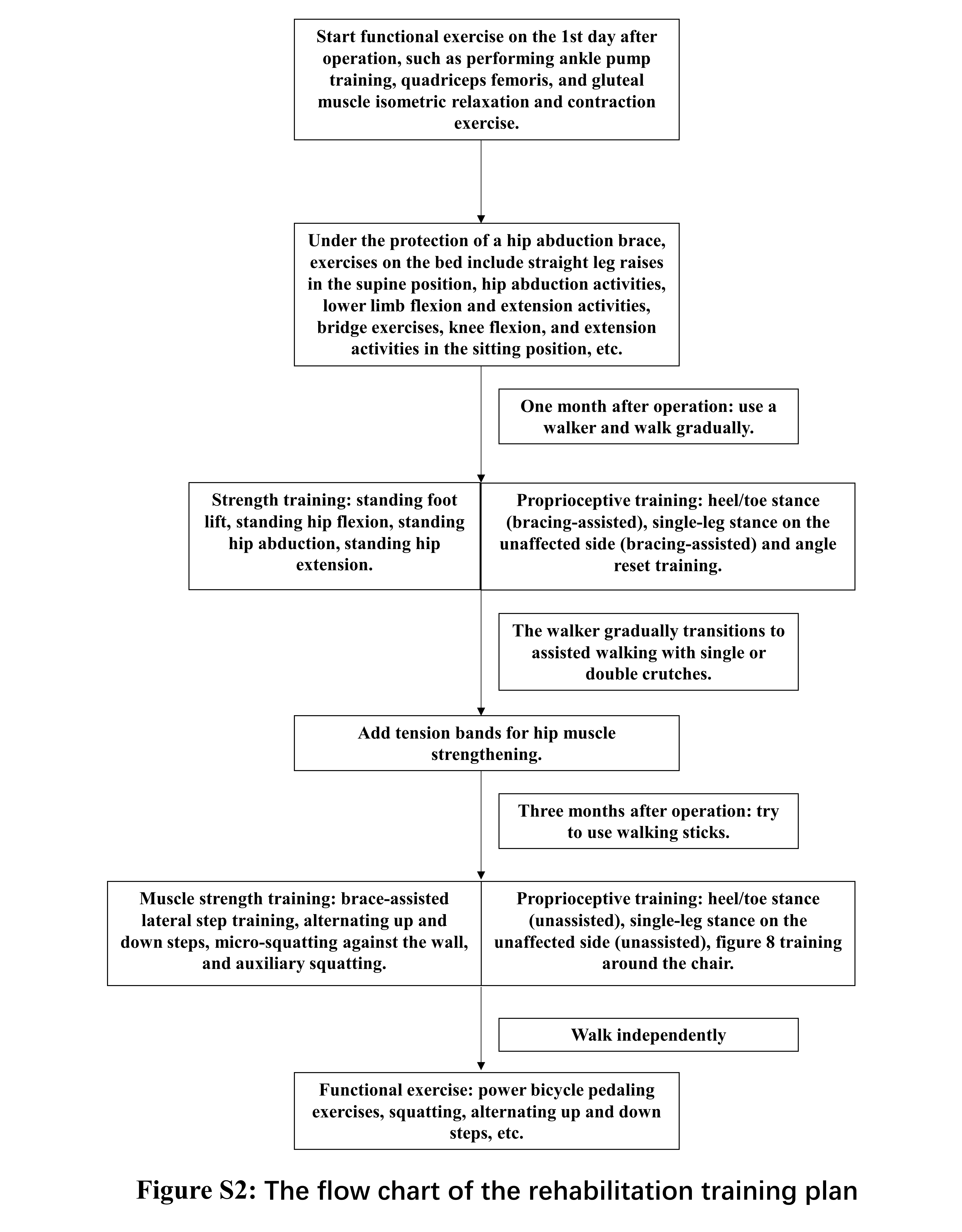

Supplement: Supplementary file 2 — Additional file 2: Figure S2. The Flow chart shows our rehabilitation training plan. [file 12893_2022_1804_MOESM2_ESM.tif]
