# Supplementary material for: 3D-printed hemipelvic prosthesis combined with a dual mobility bearing in patients with primary malignant neoplasm involving the acetabulum: clinical outcomes and finite element analysis
Source: BMC Surg. 2022 Oct 6;22:357. doi: 10.1186/s12893-022-01804-8 (PMC9541076; doi:10.1186/s12893-022-01804-8)
Supplement: Supplementary file 3 — Additional file 3: Table S1. The table shows the structural material coefficients we set in the finite element analysis. [file 12893_2022_1804_MOESM3_ESM.docx]

**Table S1: The structural material coefficients of reconstructed pelvis**

| Structural material | | Modulus of elasticity（MPa） | Poisson's ratio |
| --- | --- | --- | --- |
| Sacroiliac cortical bone | 170000 | | 0.3 |
| Sacroiliac cancellous bone | 800 | | 0.2 |
| pubic symphysis cartilage | 5 | | 0.45 |
| Sacroiliac cartilage | 11.85 | | 0.45 |
| Femoral cortical bone | 155000 | | 0.28 |
| Femoral cancellous bone | 389 | | 0.3 |
| Femoral head cartilage | 11.85 | | 0.45 |
| Acetabulum cartilage | 11.85 | | 0.45 |
| Artificial femoral head prosthesis (Delta ceramics) | 358000 | | 0.3 |
| Femoral stem prosthesis (Ti6Al4V) | 110000 | | 0.3 |
| Polyethylene liner (HXLPE) | 1000 | | 0.46 |
| Hemipelvic prosthesis (Ti6Al4V) | 110000 | | 0.3 |
| Locking screw, cancellous bone screw (Ti6Al4V) | 110000 | | 0.3 |
